# Contraceptive use and pregnancy rates among women receiving antiretroviral therapy in Malawi: a retrospective cohort study

**DOI:** 10.1186/s12978-017-0440-0

**Published:** 2018-02-09

**Authors:** Hannock Tweya, Caryl Feldacker, Salem Gugsa, Sam Phiri

**Affiliations:** 1 The International Union Against Tuberculosis and Lung Disease, Paris, France; 2 Lighthouse Trust, P.O. Box 106, Lilongwe, Malawi; 3 International Training and Education Center for Health, University of Washington, Seattle, WA USA; 4 Department of Medicine, University of North Carolina School of Medicine, Chapel Hill, NC USA; 5 Department of Public Health, College of Medicine, School of Public Health and Family Medicine, University of Malawi, Blantyre, Malawi

**Keywords:** Family planning services, Integration, Contraceptives, HIV clinical care, Antiretroviral therapy, Pregnancy rates

## Abstract

**Background:**

In 2011, family planning (FP) services were integrated at Martin Preuss Centre (MPC), in urban Lilongwe, Malawi. To date, no previous study evaluated pregnancy rates among HIV-positive women after the integration of FP services into HIV care at the facility. In this study, we investigated whether integration of FP services into HIV clinical care led to increased use of contraceptives and decreased pregnancy rates.

**Methods:**

This was a retrospective cohort analysis of HIV-positive women from 15 to 49 years of age who accessed antiretroviral therapy (ART) services at MPC. Ascertainment of FP needs, contraceptive methods and pregnancy status were done at ART initiation, and at each ART follow-up visit. Women were offered a wide range of contraceptive methods. Outcomes of interest were contraceptive use and rate of pregnancy. Incident pregnancy was ascertained through patient self-reports during clinic consultation. Trends of contraceptive use and pregnancy rates were analyzed using chi-square (χ2).

**Results:**

A total of 10,472 women were included in the analysis and contributed 15,700 person-years of observation. Contraceptive use among all women receiving ART increased from 28% in 2012 to 62% in 2016 (*p* < 0.001). A total of 501 pregnancies occurred, including 13 multiple pregnancies, resulting in an overall pregnancy rates of 3.2 per 100 person-years. Rates of pregnancy decreased from 6.8 per 100 person-years in 2012 to 1.3 per 100 person-years in 2016 (*p* < 0.001).

**Conclusion:**

Integration of FP services into HIV care resulted in increased contraceptive use and, subsequently, decreased pregnancy rates in women receiving ART. HIV programs should consider offering FP services to women who are receiving ART.

## Plain English summary

Women living with HIV appear to have a higher rate of unintended pregnancy (51–90%), compared to global estimates of other women (38%). Most frequently, family planning services are not integrated into HIV services; therefore, HIV-positive women typically must visit separate HIV clinics and reproductive health clinics to access both services. Integrating family planning services into HIV care may address some challenges faced by HIV-infected women in accessing their preferred contraceptive methods.

In 2011, family planning (FP) services were integrated into HIV care at Martin Preuss Centre (MPC) in urban Lilongwe, Malawi. In this study, we analyzed data collected during antiretroviral therapy (ART) patient visits at the HIV clinic. The study demonstrated that integrating family planning services into HIV care led to an increased use of contraceptives from 28% in 2012 to 62% in 2016 among women receiving ART and pregnancy rates decreased by 66%.

With the demonstrated reduction in pregnancy rates, the evidence supporting the need to integrate family planning services into HIV care is strong. HIV programs should consider providing reproductive health services to HIV-positive women.

## Background

In 2016, an estimated 14.8 million women were living with HIV in sub-Saharan Africa [1]. With a total fertility rate of 4.9 births per woman, sub-Saharan Africa has the highest fertility rates in the world [2]. Approximately one-quarter of women in sub-Saharan Africa do not use modern contraceptives [3]. In Malawi, specifically, the total fertility rate is 4.4 births per woman; about 41% of pregnancies are unintended [4]. Unintended pregnancies are a public health issue. Among HIV-positive women, unintended pregnancy increases the risk of vertical transmission of HIV [5], HIV-related maternal morbidity [6] and mental health problems [7]. Although some gains have been made to prevent unintended pregnancy, HIV-positive women still face challenges in accessing family planning (FP) services. Usually, FP services are mostly provided in clinics that are not connected to HIV clinics, therefore, HIV-positive women typically visit separate HIV clinics and reproductive health clinics to access both services.

Providing FP services within HIV clinical care offers an opportunity to address unmet contraceptive needs and reduce the risk of unintended pregnancy among women living with HIV [8, 9]. Adding to the growing body of evidence for the need to integrate FP services into HIV care, a study at Martin Preuss Centre (MPC) in urban Malawi reported an overall pregnancy incidence of 9.3/100 person-years between 2007 and 2010, and showed how the total fertility rate among HIV-infected women reverted to that of the average urban population in Malawi after 6 months on antiretroviral therapy (ART) (total fertility rate 3.9) [10]. MPC introduced provider-initiated FP service following the implementation of the 2011 Malawi integrated clinical HIV guidelines [11]. Accordingly, MPC began to routinely provide condoms to all adults receiving ART and offer at least a standard injectable contraceptive (depot medroxyprogesterone Acetate (DMPA)) during ART visits to women. Women who preferred other contraceptives were referred to a neighboring, external reproductive health unit for contraceptives. Efforts to implement more integrated FP services within MPC were initiated in early 2015, resulting in an array of FP methods (including oral contraceptive pills, DMPA, copper intrauterine devices, etc.) being offered within the ART clinic.

To date, no study evaluated pregnancy rates among HIV-positive women after the integration of FP services at MPC. This retrospective cohort study investigated pregnancy rates and contraceptive use among HIV-infected women accessing ART at the clinic. We compared pregnancy rates among women on ART to pregnancy rates observed in urban women in the country as whole.

## Methods

### Study design, population and setting

This retrospective cohort study used routine programme data collected at a public ART clinic, MPC, at Bwaila Hospital in urban Lilongwe, Malawi. All HIV-infected women aged 15 to 49 years who initiated ART between January 2012 and December 2016 were included in the study. We excluded (i) all HIV-positive women who registered for HIV care, but did not start ART, due to inconsistent pregnancy information among pre-ART patients, (ii) women who had less than 1 month of ART follow-up and (iii) women who were pregnant at ART registration and were lost from care within 3-months after delivery**.**

The clinic uses a real-time, point of care, electronic medical record system (EMRs) for management of HIV-positive people who are receiving ART [12]. Data for this analysis were obtained from the EMR system at MPC. In December 2016, MPC had approximately 22,000 HIV-positive people on ART (10,887 (49%) women of reproductive age) with an average of 450 attendances each day.

### Data collection through EMRs

All HIV-infected individuals who reported to the ART clinic were registered for HIV care in the EMRs. During the study period, patients were initiated on ART based on WHO clinical staging (stages 3 or 4) or CD4 cell count measurement below certain thresholds if they had WHO stages 1 or 2 conditions [11, 13]. CD4 cell count threshold for ART initiation changed over the study period, following these guidelines: ≤ 350 cells/μl between 2012 and 2013 and CD4 cell counts ≤ 500 cells/μl from 2014 to June 2016 [11, 13]. Starting July 2016, Malawi’s HIV program implemented a test and treat policy that recommended ART initiation for HIV-infected individuals regardless of any clinical or immunological status [14]. Once initiated on ART, routine visits were scheduled monthly during the first six months on ART and every two or three months thereafter if there were no clinical complications and adherence was good. Self-reported pregnancy status was ascertained at ART initiation, and then at each ART follow-up clinic visit. FP needs and contraceptive methods were recorded in the EMRs at each clinic visit.

ART program outcomes were recorded at every visit and classified as “alive on ART”, “died”, “loss to follow-up” (LTFU) (missed a clinic appointment by at least two months), “transfer-out” and “stopped ART”.

### Data management and statistical analysis

Contraceptive use was defined as using any form of contraceptive at the time of ART clinic visit including condoms and traditional methods. Women who were not sexually active were included in the denominator because (i) sexual activity status may change within a short time and (ii) women might have falsely reported not being sexually active due to the cultural sensitivity of discussing their sexual life. Use of long-acting reverse contraceptives (intrauterine devices and implants) and permanent contraceptives (tubal ligation and vasectomy) were classified as “more effective contraceptives” [15] and other contraceptives were condoms, DMPA and oral contraceptives.

The primary outcome was incident pregnancy through patient self-reports during clinic consultation. Incident pregnancy was defined as either an episode of pregnancy reported during ART treatment for women who were not pregnant at ART initiation, or any subsequent episodes of pregnancy for women who were pregnant at ART initiation. Regardless of their contraceptive use, women entered into the observation period at ART initiation if they were not pregnant at ART initiation or 3 months after delivery if they were pregnant at ART initiation. Women who transferred their HIV care from another facility were considered to come under observation when they first registered at MPC. Follow-up ended either at the time of death, LTFU or the censoring date (31st December 2016).

Descriptive statistics were used to report contraceptive use and pregnancy rates. Trends of contraceptives use and pregnancy were analyzed using chi-square (χ2). Total fertility rate (TFR) of the study population was compared to the TFR in women in urban general population as estimated by the 2015–2016 Malawi Demographic Health Survey (MDHS) [4]. According to MDHS, TFR is defined as the number of children who would be born per woman if she was to pass through the childbearing years bearing children according to a current schedule of age-specific fertility rates. We used TFR rather than standardized fertility rate because it is the most widely used measure of fertility and unaffected by age composition.

## Results

Of 13,565 women who accessed ART services at MPC, 3093 (23%) were excluded from the study because their ART follow-up was less than 1 month or they were pregnant at ART registration and were lost from HIV care within 3-month after delivery. A total of 10,472 (77%) women were included in the analysis and contributed 95,644 clinical visit observations. Median age at ART initiation was 29 [inter-quartile range (IQR): 24–34] years. At ART initiation, 7597 (75%) had WHO clinical stage 1 or 2 conditions; 2113 (21%) had stage 3 conditions; 475 (4%) had stage 4 conditions; and 287 (3%) had no WHO clinical staging information (Table 1). Of the 2588 (25%) with baseline CD4 cell count data, the median CD4 count at ART initiation was 235 cells/mm3 (IQR: 132–332).

**Table 1:** Baseline characteristics of women who accessed ART at Martin Preuss Centre in Lilongwe, Malawi, between January 2012 and December 2016

Characteristics	Women: # (%)		Number of pregnancies	Observed rate per 100 person-years (95% confidence interval)	
Age ART initiation^a^					
15–19	734	(7%)	41	4.4	(3.3–6.0)
20–24	2151	(21%)	144	5.1	(4.3–6.0)
25–29	2806	(27%)	180	4.2	(3.7–4.9)
30–34	2384	(23%)	98	2.6	(2.2–3.2)
35–39	1328	(13%)	32	1.4	(1.0–2.0)
40–44	701	(7%)	5	0.4	(0.2–1.0)
45–49	325	(3%)	0	0	–
WHO clinical stage at ART initiation^b^					
1 or 2	7597	(75%)	380	3.3	(3.0–3.7)
3	2113	(21%)	90	2.8	(2.3–3.4)
4	475	(4%)	24	2.8	(1.9–4.3)
CD4 count (cells/mm3) at ART initiation^c^					
< 250	1390	(54%)	81	3.1	(2.5–3.8)
250 to 349	662	(26%)	43	3.4	(2.5–4.6)
≥ 350	536	(21%)	14	2.2	(1.3–3.8)

^a^43 women were less than 15 years at ART initiation; ^b^287 women had no WHO clinical stage at ART initiation; ^c^7,884 women had no CD4 count at ART initiation

### Contraceptive use

Trends in contraceptive use were analyzed by calendar year of implementation of FP services and follow-up time. Contraceptive use among all women receiving ART increased from 28% in 2012 to 62% in 2016 (*p* < 0.001) (Table 2). At baseline, 5368 (51%) women were assessed for current FP use: 1679 (31%) indicated using contraceptives at baseline. Of those, 800 (50%) reported using DMPA, 74 (5%) oral contraceptives, 458 (29%) more effective methods and 262 (16%) condoms. Information on contraceptive methods was missing for 85 (5%) women. At two years of observation, 1810 (60%) were using contraceptive methods; 759 (42%) were using DMPA, 51 (3%) oral contraceptives, 490 (27%) other effective methods and 365 (20%) condoms. Contraceptive methods were not recorded in 145 (8%) women.

**Table 2:** Contraceptive use and pregnancy rates by calendar years, among HIV-infected women receiving ART at Martin Preuss Centre, Lilongwe, Malawi (*N* = 10,472)

	2012	2013	2014	2015	2016	*P*-value for trend
Number of women	1590	3295	4666	5925	7463	
Clinic review visits	7663	15,687	21,297	26,474	24,523	
Assessment of contraceptive use	6615 (86%)	13,590 (87%)	18,174 (85%)	22,103 (83%)	22,008 (90%)	
Contraceptive use	28%	49%	59%	61%	62%	< 0.001
More effective contraceptives^a^	26%	26%	28%	28%	27%	< 0.001
Pregnancy rates /100 person-years	6.8 (5.2–8.8)	4.1 (3.4–5.1)	4.0 (3.4–4.7)	3.3 (2.8–3.8)	1.3 (1.0–1.7)	

^a^More effective contraceptive methods are implants; intrauterine devices, tubal ligation and vasectomy

### Pregnancy rates

**Fig. 1:**
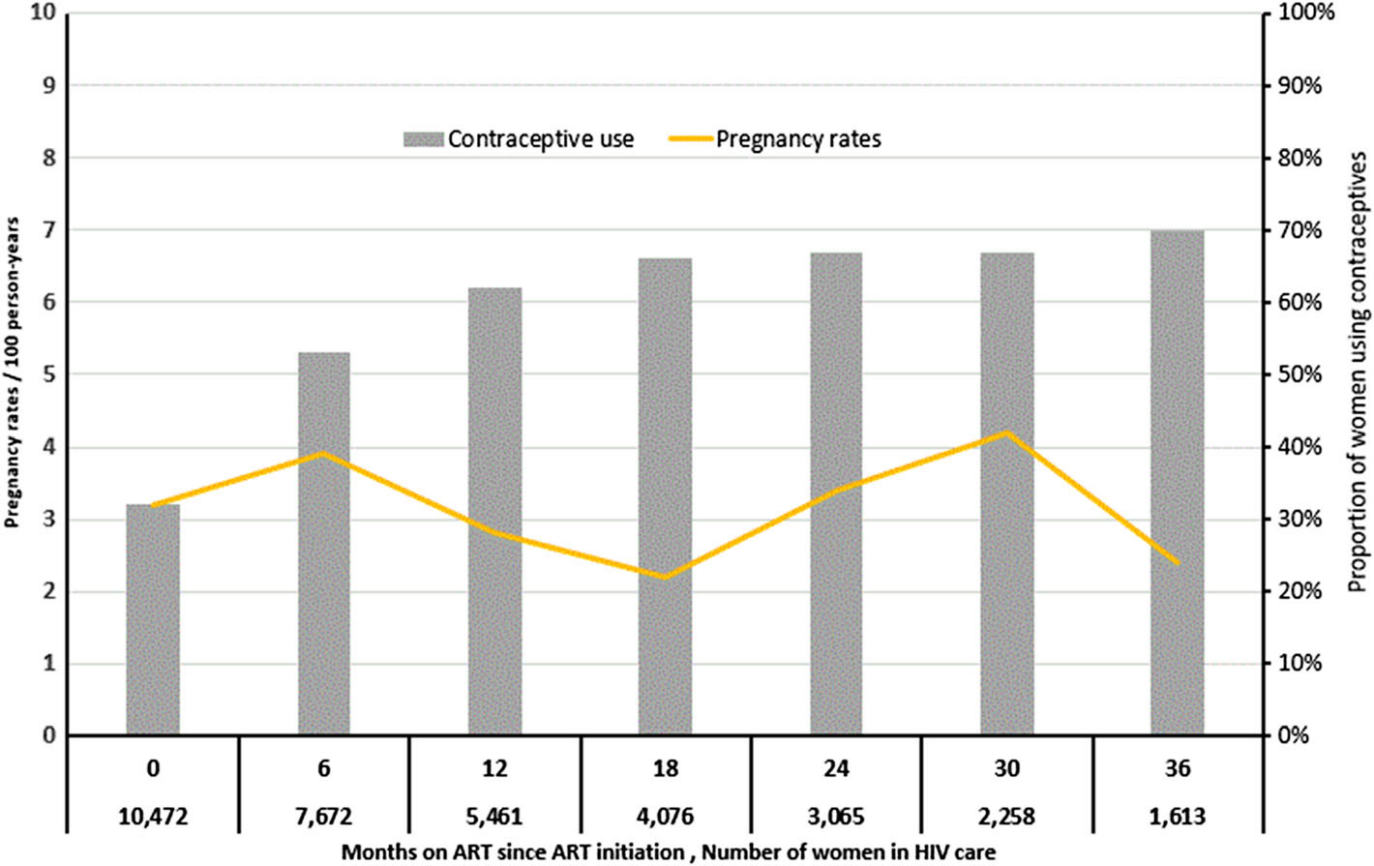
Contraceptive use and pregnancy rates during follow-up among HIV-infected women receiving ART at Martin Preuss Centre, Lilongwe, Malawi

A total of 501 pregnancies occurred, including 13 multiple pregnancies, during 15,700 person-years of observation. Overall pregnancy rate was 3.2 per 100 person-years of observation time. Pregnancy rates decreased from 6.8 per 100 person-years in 2012 to 1.3 per 100 person-years in 2016 (*p* < 0.001) (Table 2). Women who became pregnant after starting ART were younger than their non-pregnant counterparts (median age 26 vs. 29 years; *p* < 0.001). During follow-up time, pregnancy rates remained stable at 3.1 per 100 person-year (Fig. 1). However, cumulative pregnancy rates at 6-, 12-, 24- and 36- months of observation were 1.6% (95% CI 1.29–1.96), 3.5% (95% CI 3.09–4.05), 6.2% (95% 5.57–6.90) and 10% (95% CI 9.07–11.04), respectively. Overall, the TFR of women on ART (TFR 0.9) was lower than TFR of women in the general urban population (TFR 3.0).

## Discussion

In this retrospective cohort study, contraceptive use increased with time of follow-up and increasing year of implementation of integrated FP services. The high, post-intervention contraceptive use resulted in a lower overall pregnancy rates of 3.2 per 100 person-years compared to 9.3 per 100 person-years reported in the pre-intervention study [10]. Pregnancy rates decreased steadily during the five-year study period. The TFR of women in the study was also lower than both the TFR of the general population of urban Malawian women [4] and the TFR among pre-intervention women in 2010 (TFR 3.1) [10], suggesting the impact of the integrated, comprehensive FP services in HIV-infected women.

Overall fertility rates declined in Malawi over the past six years: the TFR decreased by 25% between 2010 and 2016 among urban population women in Malawi [4]. Although some of the drop in TFR observed in the study cohort may likely be due to population-level trends, the steep TFR decrease of 71% among study women cannot be entirely explained by societal factors, alone. Rather, the comprehensive integration of FP services into HIV care may have complemented women’s increased desire to use contraceptives methods. A third of women in the study were using contraceptives at baseline; however, the proportion of women that used contraceptives over follow-up period peaked to 62%, suggesting that repeated counseling on FP may have improved use of contraceptive. Provision of FP within HIV clinical care may also have addressed some challenges faced by HIV-infected women in accessing their preferred contraceptive methods. The provision of FP and HIV-related services in one clinic visit also minimizes transportation costs and wait times that a patient would have incurred during multiple clinic visits. Given the high uptake of contraceptives in women receiving ART, public HIV clinics should consider integrating comprehensive family planning services in order to effectively reduce unintended pregnancies.

We found an overall contraceptive use of 62% in 2016. As compared to our result, some recent studies among women receiving ART in Ethiopia, Nigeria and Ghana reported a lower contraceptive rate, 44%, 36% and 43%, respectively [16–18], while a study in Zambia reported higher contraceptive use of 69% [19]. Unlike our study, however, family planning services were not fully integrated in HIV clinical care in these studies and most preferred methods were male condoms, except in Ethiopia. These comparisons highlight variations of contraceptive service delivery and contraceptive uptake among HIV-positive women who are receiving HIV-related care in sub-Saharan Africa.

Although our findings are similar to a South African study that reported pregnancy rates of 3.95 per 100 person-years [20], it contrasts with other recent studies reporting higher rates of pregnancy among women on ART in sub-Saharan Africa [21, 22]. These contrasts may result from the differences in the availability of FP services or the level of FP integration into HIV services. At MPC, several steps were taken to ensure the successful integration of FP services in HIV clinical care. First, patients were informed about availability of FP services through both oral and visual methods, increasing their awareness of FP services through daily patients’ education sessions. Second, providers were well trained in reproductive health services. Their clinical service provision was also supported by EMRs that guided service provision and clinical decisions during each clinic visit. These steps, which may not have been applied elsewhere, likely led to the attainment of full FP integration at MPC.

This study has several limitations. First, the assessment of pregnancy was based upon self-reports, which is known to be affected by social desirability bias and depended on whether the women knew they were pregnant or not at the time of the clinic visit. Second, this study used routine data from the HIV clinical care and some information was not captured in EMRs due to unavailability of electrical power at the time of the clinic visit. Information on contraceptive use was not available in 13% of the women that were supposed to be assessed. Lastly, use of EMRs combined with sound management and implementation efforts to prepare providers on FP provision and encourage client uptake at MPC may not be easily applied elsewhere, potentially decreasing the success of FP integration in other settings. Despite these limitations, our findings support the provision of comprehensive FP services to HIV-infected women can reduce unintended pregnancies.

## Conclusion

The improved FP access and availability due to FP integration into HIV care led to increased contraceptive uptake and, subsequently, decreased pregnancy rates among HIV-infected women receiving ART. Other high HIV prevalence settings in sub-Saharan Africa should consider integrating FP into routine HIV care to improve reproductive health outcomes.

## Abbreviation

ART: Antiretroviral therapy
DMPA: Depot Medroxyprogesterone Acetate
EMRs: Electronic Medical record system
FP: Family planning
HIV: Human Immunodeficiency Virus
MDHS: Malawi Demographic Health Survey
MPC: Martin Preuss Centre
TFR: Total fertility rate
WHO: World Health Organization
